# Application of FF-QuantSC for the Precise Estimation of Fetal Fraction in Non-invasive Prenatal Testing in Two SRY-Translocation Cases

**DOI:** 10.3389/fgene.2020.570333

**Published:** 2020-10-14

**Authors:** Yan Zeng, Jiong Gao, Hua Yuan, Lijun Zhou, Dehua Cheng, Ming Che, Yandi Qian, Jiaming Fan, Lifang Zhang, Feiyan Qian, Yuling Gao, Tingting Luo, Weiping Chen, Ting Wang, Yaoxiang Jin, Jian Zhao, Xiaoliang Shi, Hongmei Li, Haitao Pan, Cheng Xiong, Yunqin Ni, Shuchao Qiu, Tao Zhang

**Affiliations:** ^1^Shaoxing Maternity and Child Health Care Hospital, Shaoxing, China; ^2^Clinical Laboratory of BGI Health, BGI-Shenzhen, Shenzhen, China; ^3^Reproductive and Genetic Hospital of CITIC-Xiangya, Changsha, China; ^4^Obstetrics and Gynecology Hospital of Shaoxing University, Shaoxing, China

**Keywords:** non-invasive prenatal testing, fetal fraction, Y chromosome, FF-QuantSC, SRY translocation

## Abstract

**Background**: Non-invasive prenatal testing (NIPT) is a commonly employed clinical method to screen for fetal aneuploidy, while the Y chromosome-based NIPT method is regarded as the gold standard for the estimation of fetal fraction (FF) of male fetuses. However, when the fetus has a derivative Y chromosome thereby containing a partial Y chromosome, the Y chromosome-based NIPT method cannot accurately calculate FF. Therefore, alternative methods to precisely calculate FF are required.

**Methods**: Two prenatal cases could not be detected effectively using the Y chromosome-based NIPT method because of low FF. According to the Y chromosome-based method, the FF of the fetuses were 1.730 ± 0.050% (average gestation week: 18^+1^) and 2.307 ± 0.191% (average gestation week: 20^+0^) for cases 1 and 2, respectively. Using various genetic diagnostic techniques, including the BoBs™ assay, karyotype analysis, improved nucleolus-organizing region (NOR)-banding analysis, Affymetrix CytoScan 750K Array, and fluorescence *in situ* hybridization (FISH) analysis, we determined the genetic defects of two fetuses with translocations of the SRY locus. Further, we reassessed the FF using FF-QuantSC and X chromosome-based methods. The distribution diagram of reads for chromosome Y was also analyzed.

**Results**: The FF of the fetuses determined by FF-QuantSC were 10.330% (gestation week: 18^+4^) in case 1 and 9.470% (gestation week: 21^+4^) in case 2, while the FF of the fetuses determined using the X chromosome-based method were 8.889% (gestation week: 18^+4^) in case 1 and 2.296% (gestation week: 21^+4^) in case 2. Both the distribution diagrams of reads for chromosome Y of the two cases showed the deletion in the long arm of the Y chromosome.

**Conclusion**: For repeatedly low FF samples detected using the Y chromosome-based NIPT method for a long gestational week, we believe that FF-QuantSC and distribution diagrams of reads could be used as a supplement to NIPT, especially for rare cases of sex reversal caused by SRY translocation.

## Introduction

Since cell-free fetal DNA (cffDNA) was detected in cell-free DNA (cfDNA) obtained from the plasma of pregnant women (Lo et al., 1997), comparison of chromosome dosage distribution of cffDNA between patients and controls has played an increasingly important role in the diagnosis of fetal aneuploidy (Wang et al., 2006). Statistics show that approximately one in every 150 live births harbors chromosomal abnormalities and roughly one in every 800 live births exhibits Down syndrome (trisomy 21; Gregg et al., 2016). As an alternative screening method, non-invasive prenatal testing (NIPT) has proven to be highly sensitive and specific for the detection of common chromosomal aneuploidies, such as trisomy 21, trisomy 18, and trisomy 13, with low false-positive and false-negative rates (Song et al., 2013; Bianchi et al., 2014; Chandrasekharan et al., 2014; Liao et al., 2014). A recent NIPT study on chromosomal aberrations showed that NIPT has high performance for copy number variations (CNVs) in the first trimester and that it can be adopted as a first-tier prenatal approach (Cui et al., 2019; Liang et al., 2019). NIPT evaluates the risk of fetal chromosomal aneuploidies by detecting cffDNA circulating in maternal plasma *via* next-generation sequencing (NGS) technology (Chandrasekharan et al., 2014; Xue et al., 2019).

Fetal fraction (FF), the fundamental parameter of NIPT, is the proportion of cffDNA in maternal plasma (Canick et al., 2013). Numerous studies have demonstrated that an FF lower than 5% is unreliable and that the fluctuation of FF significantly impacts the accuracy of NIPT screening by elevating false-positive and false-negative rates (Canick et al., 2013; Takoudes and Hamar, 2015; Zhang et al., 2015; Tian et al., 2018). To date, studies have shown that gestational length and maternal weight influence FF (Wataganara et al., 2004; Wang et al., 2013; Kinnings et al., 2015). Each method for FF detection has limitations that are yet to be overcome. The methylation-based method requires whole-genome bisulfite sequencing, while the size-based method calls for the sequencing of paired-ends. Both sequencing methods increase the cost of routine NIPT. Although the method based on single-nucleotide polymorphisms (SNPs) is accurate, it requires additional parental SNP information, which may not be easily obtained. Moreover, the Y chromosome-based method is not suitable for female fetuses; however, it was regarded as the gold standard for the estimation of FF in male fetuses (Yuan et al., 2020). When a fetus has a derivative chromosome containing a partial Y chromosome, FF cannot be accurately measured using Y chromosome-based NIPT. Recently, a new FF estimation method that can circumvent these limitations has been developed. The method, FF-QuantSC, is known for accurate quantification of FF with shallow-coverage sequencing of maternal plasma DNA and it employs an artificial neural network model (Yuan et al., 2020), which can avoid the inaccuracy of FF value caused by chromosome abnormality, especially sex chromosome abnormality.

In our study, a derivative chromosome containing a partial Y chromosome could not be detected effectively using the Y chromosome-based NIPT method in two prenatal cases owing to low calculated value of FF. Case 1 was a fetus with 45, X, dic (Y; 21; q11; p11), ish dic (Y; 21; SRY+, CEPY+, CEP21+). Aberrant chromosome 21 in the fetus’s karyotype had a significantly longer short arm in G bands as observed following trypsin using Giemsa stain (GTG)-banding and no silver stain in terms of nucleolus-organizing region (NOR)-banding. The mother had a similar chromosome 21 with a longer short arm with respect to GTG banding; however, NOR-banding showed a large silver stain in the short arm of chromosome 21. The father had a normal Y chromosome structure with 46, XY, ish X (DXZ1 × 1), ish Y (SRY × 1), as assessed by fluorescence *in situ* hybridization (FISH). FF was 1.776, 1.677, and 1.738% at gestation weeks 17^+1^, 18^+4^, and 18^+4^, respectively. After recalculating FF using FF-QuantSC, the actual FF was 10.330% at gestation week 18^+4^. Case 2 was a fetus with 46, XX, ish X (DXZ1 × 2, SRY × 1) and the father had a normal Y chromosome structure as determined by FISH. FF was 2.177, 2.218, and 2.526%, at gestation weeks 19^+4^, 21^+4^, and 21^+4^, respectively. After recalculating FF using FF-QuantSC, the actual FF was 9.470% at gestation week 21^+4^. The distribution diagrams of reads for chromosome Y indicated that both cases lacked the long arm of the Y chromosome. Based on these cases, we established a standard FF-detection process that will facilitate positive detection rate of NIPT and reduce false-negative rates.

## Materials and Methods

### Editorial Policies and Ethical Considerations

The study was carried out with the authorization of the Hospital Ethics Committee of the Shaoxing Maternity and Child Health Care Hospital and the Ethics Committee of the Clinical Laboratory of BGI Health. The participating pregnant women signed informed consent forms before sample collection and agreed that the sequencing data could be used for research after anonymization.

### Collection and Treatment of Blood Samples

All women were of 12–24 weeks (17.62 ± 4.07) after gestation at the time of sample collection. Maternal peripheral blood samples (5 ml) were collected in cfDNA storage tubes (CW2613S; BGI, Shenzhen, China), thoroughly mixed, and stored temporarily at 6–35°C. The blood samples (stored for a maximum of 3 days) were then centrifuged at 1,500 × *g* for 10 min and the plasma was collected and dispensed into 2.0-ml Eppendorf tubes. The plasma was centrifuged again at 15,000 × *g* for another 10 min. The upper phase of the plasma was carefully divided into 600-μl portions and transferred into new 2.0-ml Eppendorf tubes and stored at −80°C before testing.

### Non-invasive Prenatal Testing Using the BGISEQ-500 Sequencing Platform

DNA extraction, library construction, and sequencing were performed according to the standard protocol of the Shaoxing Maternity and Child Health Care Hospital mentioned in the Human Molecular Genetics Guidelines. cffDNA extraction was performed with maternal plasma (200 μl) using the BGISP-300 (BGI, Shenzhen, China) and the Nucleic Acid Extraction (BGI, Shenzhen, China) kits. Next, end-repair enzymes were added and the conditions were as follows: 37°C for 10 min and 65°C for 15 min, followed by adapter ligation at 23°C for 20 min with label-adapter and ligase. After end-repair and adapter ligation, PCR was used to amplify DNA to the desired concentration under the following cycling conditions: 98°C for 2 min, followed by 12 cycles at 98°C for 15 s, 56°C for 15 s, and 72°C for 30 s, with a final extension at 72°C for 5 min. The DNA amplification products were quantified on a Qubit® 2.0 fluorometer (Thermo Fisher Scientific, Walsham, United States) using the Qubit™ dsDNA HS Assay kit (Thermo Fisher Scientific, Walsham, United States), and a concentration ≥2 ng/μl was regarded as the minimum qualifying standard. The mass was calculated according to the concentration of each sample and all samples of the same mass were pooled together. The DNA double strands were thermally denatured into single strands after pooling, followed by the addition of the cyclic buffer and ligase to prepare circular DNA according to the cyclization reaction. The circular DNA molecules were used to make DNA nanoballs (DNBs) by rolling-circle replication (RCR). The concentration of DNBs was determined on a Qubit® 2.0 fluorometer using the Qubit™ ssDNA Assay kit (Thermo Fisher Scientific, Walsham, United States), and a DNB concentration within the range of 8–40 ng/μl was considered ideal. The DNBs were loaded onto chips and sequenced on the BGISEQ-500 sequencing platform (BGI, Shenzhen, China). Any sample that failed to meet quality control criteria was reported as a detection failure by NIPT.

### Improved Nucleolus-Organization Region-Banding Analysis

Nucleolus-organization region-banding is based on selective silver staining of regions containing clusters of functional rRNA genes. Silver staining is an important method for studying heteromorphic variation and structural rearrangements involving the acrocentric chromosome. Our improved NOR-banding method allows for unequivocal identification of chromosome pairs bearing NORs.

The traditional NOR-banding method was a simplified method following the technique of Bloom and Goodpasture (1976). Silver nitrate (0.5 g) was added to 1 ml of 1% formic acid solution to prepare the Ag-staining solution (the Ag-staining solution was prepared fresh every time) and the slide was covered with 2–3 layers of microscope lens paper as a filter. Five drops of Ag-staining solution were added to the slide and treated at 60°C for 25 min in a moist chamber, followed by rinsing with distilled water. The slide was then stained with Giemsa solution in phosphate buffer (pH 6.8) for 3 min. Under the microscope, satisfactory NOR-banding karyotypes were observed and the images were captured (the coordinates were recorded).

Our improved NOR-banding method includes three steps after the images are captured: (1) the slides were rinsed in 75% ethanol to wash out the Giemsa stain and then rinsed with saline solution; (2) the slides were treated with trypsin solution for 2–3 min, washed in saline solution, and finally stained with Giemsa solution for 3 min; and (3) according to the recorded coordinates, the second images of the same karyotype were captured for comparison. If the GTG bands of chromosomes were not clear enough to identify each chromosome, the steps were repeated.

### Fluorescence *in situ* Hybridization Analysis

Fluorescence *in situ* hybridization analysis was performed with commercially available probes for case 1 [SRY (red)/CEP 13/21 (green)] and case 2 [SRY (red)/CEP X (green)], which were purchased from Abbott Co. (Abbott Laboratories, Chicago, United States). Probe hybridization and detection were conducted according to the manufacturer’s instructions. The slides were examined with a Zeiss Imager A2 microscope (Carl Zeiss, Jena, Germany) and the Isis FISH Imaging System (MetaSystems, Altlussheim, Germany).

### FF-QuantSC General Flow

The overall analysis flow for FF-QuantSC is summarized by Yuan et al. (2020). The method used in this study is as follows (Yuan et al., 2020): (1) data preparation – sequencing reads were mapped to the reference sequence (GRCh37). The mapped reads were then filtered and counted to construct feature matrices of different datasets; (2) model training – the network model was trained by the planning procedures; and (3) model evaluation – after model training, the predictive results for all test datasets were assessed to evaluate the performance of FF-QuantSC. DNA sequencing was carried out on the BGISEQ-1000 platform, resulting in single-end reads of 28 bp. The original sequencing data were aligned with the human reference genome (GRCh37) using BWA (V0.7.7-r441; Li and Durbin, 2010). Unmatched mapping or multiple hit reads were removed. The reserved effective reads were divided into continuous genomic windows of 60 kb in length. Then, the windows showing no coverage were consequently removed and principal component analysis (PCA) for feature selection was applied. Standardization was carried out by dividing the data for each selected feature by the sum of the data for all features. The data were further normalized by z-score transformation in the sample. This generated a final feature matrix in which each row represented a sample and each column represented a selected feature. Lastly, the results from the testing sets were compared to evaluate the prediction ability of the training model.

## Results

### Fetal Fraction Detection Using the Traditional Y Chromosome-Based Method

The fluctuation of FF has a significant effect on the accuracy of NIPT screening. In order to assess the relationship between Y chromosome abnormalities and FF, we retrospectively analyzed pregnancies with available NIPT data from our hospital (Shaoxing Maternity and Child Health Care Hospital) and found two cases with low FF determined by the traditional Y chromosome-based method. The FF of the fetus in case 1 was 1.776, 1.677, and 1.738% at gestation weeks 17^+1^, 18^+4^, and 18^+4^, respectively. The FF of the fetus in case 2 was 2.177, 2.218, and 2.526% at gestation weeks 19^+4^, 21^+4^, and 21^+4^, respectively. The average FF of the fetuses was 1.730 ± 0.050% (average gestation week: 18^+1^) and 2.307 ± 0.191% (average gestation week: 20^+0^) in cases 1 and 2, respectively. Numerous studies have shown that an FF lower than 5% is unreliable. Thus, both the samples failed to meet the quality control criteria of the NIPT, resulting in sequencing failure.

### BoBs™ Assay

**Figure 1:**
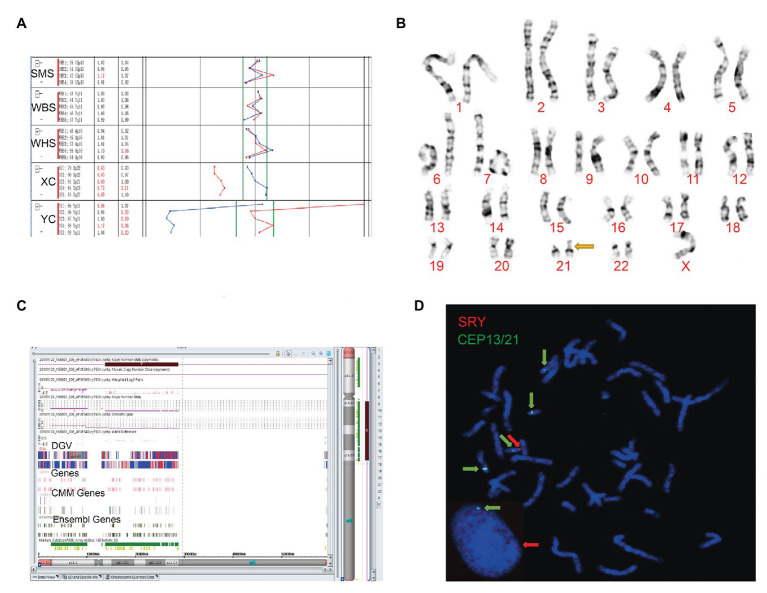
The clinical features of case 1. **(A)** Four probes in Yq11 were all absent and one probe in Yp11 presented in the BoBs™ assay. **(B)** The karyotype of the fetus (45, X). **(C)** The Affymetrix CytoScan 750K Array showed a deletion of 14.9 Mb in Yq11.21q11.23 (13,800,955–28,799,654). **(D)** Fluorescence *in situ* hybridization (FISH) showed that SRY translocated on the short arm of chromosome 21 in the fetus.

**Figure 2:**
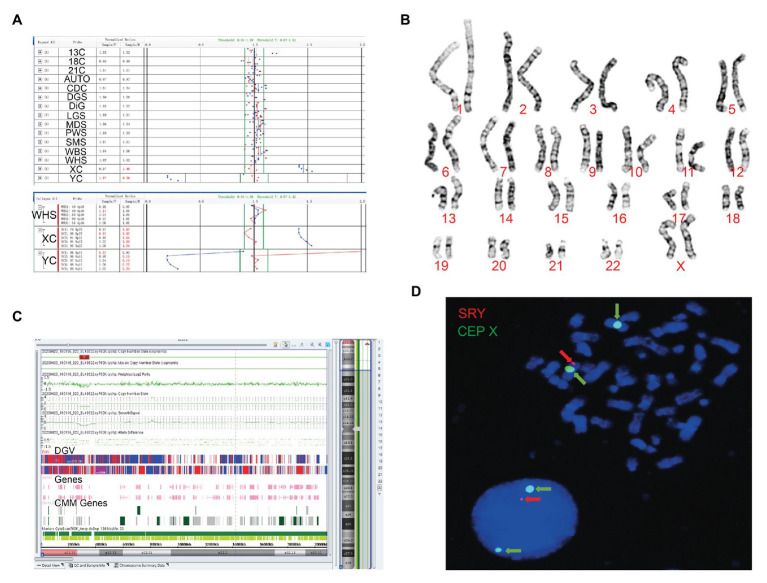
The clinical features of case 2. **(A)** Four probes in Yq11 were all absent and one probe in Yp11 presented in the BoBs™ assay. **(B)** The karyotype of the fetus (46, XX). **(C)** The Affymetrix CytoScan 750K Array showed existence of 3.7 Mb in Yp11.31p11.2 (2,650,424–6,356,292) and 2.6 Mb in Yp11.2 (7,454,508–10,073,965), and a deletion of 704.3 Kb in Xp22.33 (2,856,730–3,561,110). **(D)** FISH showed that SRY translocated on the long arm of chromosome X in the fetus.

Although no abnormal ultrasound findings were detected, both cases continued with prenatal diagnosis of the unsuccessful NIPT test. When compared to the male control, the BoBs™ assay of the fetus in case 1 showed that four Yq probes were missing (Figure 1A). In case 2, an extra Yp11 signal was observed compared to the female control (Figure 2A).

### Karyotype Analysis

Subsequently, karyotype analysis was used to further check the structure and number of chromosomes using a low-depth approach. The case 1 fetus had 45, X karyotype, with a longer short arm in one of the chromosomes of chromosome 21 (Figure 1B), but no silver stain was found for the abnormal chromosome 21 in terms of improved NOR-banding analysis (Supplementary Figure 1). Then, we checked the karyotype results of the parents. The father of the fetus in case 1 had a normal karyotype: 46, XY. The mother of the fetus in case 1 had 46, XX, with a longer short arm in one chromosome 21; however, the enhanced NOR-banding analysis featured a large silver stain in the short arm of chromosome 21, suggesting that the abnormal chromosome 21 of the fetus was not inherited from the mother. The fetus in case 2 had 46, XX (Figure 2B), and the karyotype results of the parents were normal.

### Affymetrix CytoScan 750K Array Analysis

Subsequently, the Affymetrix CytoScan 750K Array (Thermo Fisher Scientific, Walsham, United States) was used to check the chromosomes of the fetuses employing a high-depth approach. The analysis of the fetus in case 1 showed a deletion of 14.9 Mb in *Yq11.21q11.23* (13,800,955–28,799,654; Figure 1C). There were 29 Online Mendelian Inheritance in Man (OMIM) genes in the deletion, including *CDY1* (400016), *CDY2A* (400018), *DAZ1* (400003), *DAZ3* (400027), and *DAZ2* (400026). It has been reported that the deletion of the AZF region in the long arm of the Y chromosome, which contains these genes, is one of the reasons for male azoospermia, oligospermia, and infertility (Fan et al., 2002). The Affymetrix CytoScan 750K Array analysis of the fetus in case 2 showed the insertion of 3.7 Mb in *Yp11.31p11.2* (2,650,424–6,356,292) and another 2.6 Mb in *Yp11.2* (7,454,508–10,073,965), and a deletion of 704.3 Kb in *Xp22.33* (2,856,730–3,561,110; Figure 2C). There were five OMIM genes in the region of 3.7 Mb in *Yp11.31p11.2* (2,650,424–6,356,292), including *SRY* (480000), *RPS4Y1* (470000), *ZFY* (490000), *TGIF2LY* (400025), and *PCDH11Y* (400022). Mutation/deletion of the *SRY* gene is associated with Y-linked 46, XY sex reversal (46, XY sex reversal 1) disease (Ayesha et al., 2012; Sezgin et al., 2013; Yang et al., 2018; Chen et al., 2019). There was one OMIM gene in the region of 2.6 Mb in *Yp11.2* (7,454,508–10,073,965), *TSPY1* (480100). Since these studies, there have been no clear reports regarding *TSPY1* gene-related diseases, and gene repetition could be a benign mutation. There were five OMIM genes in the 704.3 kb deletion of *Xp22.33* (2,856,730–3,561,110), including *ARSE* (300180). The mutation/deletion of the *ARSE* gene is related to an X-linked recessive disorder of chondrodysplasia punctata (X-linked receptive). The clinical phenotypes include hypoplasia of the phalanx, hypoplasia of the nasal bone, hypoplasia of the punctate cartilage, and mental disorders (Michelle et al., 2008).

### Fluorescence *in situ* Hybridization Analysis

Based on the BoBs™ assay, karyotype analysis, improved NOR-banding analysis, and Affymetrix CytoScan 750K Array, we analyzed the inheritance in cases 1 and 2. In order to decipher the chromosomal structure, we applied FISH analysis to assess the fetal karyotype of both fetuses. The final fetal karyotype based on FISH in case 1 was 45, X, dic (Y; 21; q11; p11), ish dic (Y; 21; SRY+, CEPY+, CEP21+; Figure 1D). For case 2, the final fetal karyotype based on FISH was 46, XX, ish X (DXZ1 × 2, SRY × 1; Figure 2D).

### Recalculated Results for Fetal Fraction Using the FF-QuantSC and X Chromosome-Based Methods

**Figure 3:**
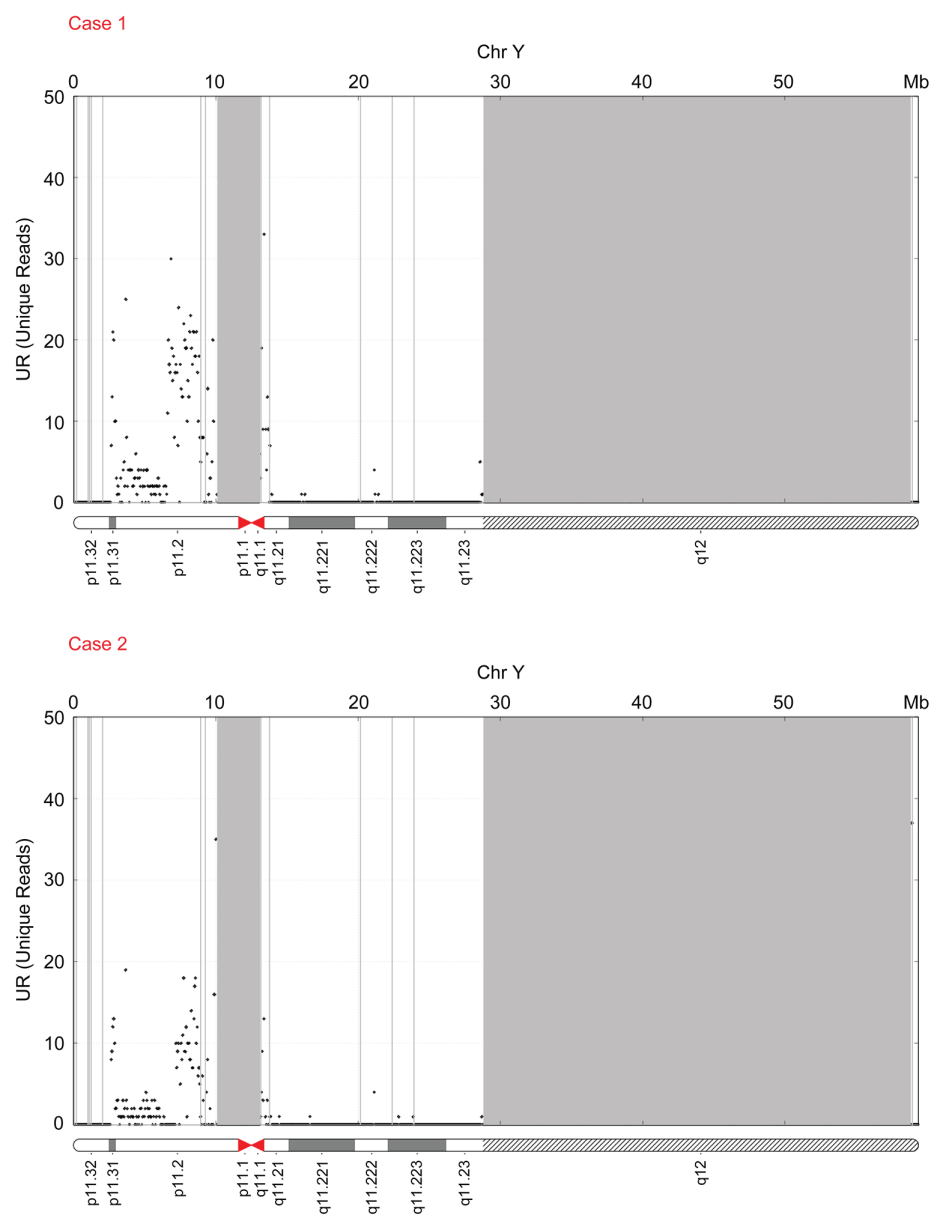
The distribution diagram of reads in chromosome Y of case 1 and case 2.

FF-QuantSC employs an artificial neural network model and can compensate for the shortcomings of the traditional Y chromosome‐ and X chromosome-based methods for the calculation of the FF/cffDNA ratio. The FF of the fetuses was 1.730 ± 0.050% (average gestation week: 18^+1^) and 2.307 ± 0.191% (average gestation week: 20^+0^) for cases 1 and 2, respectively, using the Y chromosome-based method. Next, we recalculated the FF using FF-QuantSC and the X chromosome-based methods. The actual FF of the fetuses in FF-QuantSC was 10.330 (gestation week: 18^+4^) and 9.470% (gestation week: 21^+4^), respectively. The FF of the fetuses with the X chromosome-based method was 8.889 (gestation week: 18^+4^) and 2.296% (gestation week: 21^+4^) for cases 1 and 2, respectively, which was significantly lower than the value calculated using FF-QuantSC. These results are illustrated in the distribution diagram of reads for chromosome Y (Figure 3).

## Discussion

China is a country with a high incidence of birth defects, approximately 5.60%, and the number of birth defects is around 900,000 every year as reported in the Prevention and Treatment of Birth Defects in China. Moreover, birth defects have gradually become the main cause of infant death and child disability (Cheung et al., 2005). With the development of whole-genome sequencing technology for cfDNA in maternal plasma, cfDNA screening has become an established method for the detection of fetal aneuploidy (Cuckle et al., 2013). The clinical application of NIPT has been recognized as an efficient screening test for common autosomal aneuploidies (trisomy 13, 18, and 21; Luo et al., 2020). FF, an important parameter of NIPT detection, is the proportion of cfDNA from the fetus. However, the accurate determination of FF has always been a major hurdle in NIPT. In this study, we focused on two fetuses with translocations of the SRY locus. The FF of the fetuses was 1.730 ± 0.050% (average gestation week: 18^+1^) and 2.307 ± 0.191% (average gestation week: 20^+0^) as calculated using the Y chromosome-based method. The two samples failed to meet the quality control criteria of NIPT because of the low FF values, thereby resulting in sequencing failure. FF-QuantSC accounts for the characteristics of samples with moderate FF during artificial neural network training, thus avoiding the fluctuation caused by chromosome abnormality in the samples. After recalculating the FF using FF-QuantSC, the FF of the fetuses in cases 1 and 2 were 10.330 (gestation week: 18^+4^) and 9.470% (gestation week: 21^+4^), respectively. Interestingly, the FF of the fetuses calculated using the X chromosome-based method was 8.889 (gestation week: 18^+4^) and 2.296% (gestation week: 21^+4^), respectively, which was significantly lower than the value calculated using FF-QuantSC. In addition, FF-QuantSC has potential application in the analysis of samples obtained from twins; however, it requires extensive clinical research. Moreover, we tried to modify the FF of these two cases using the derivation method. The long arm of the Y chromosome is about four times the length of the short arm. Based on the deletion in the long arm, the value of FF in the Y chromosome-based method may be reduced to 20% (1/5). Therefore, we increased the FF value five times. Consequently, the FF value in case 1 reached 8.650 ± 0.250% and the FF value in case 2 reached 11.535 ± 0.955%, which were close to the FF values calculated using FF-QuantSC.

In addition, we established a set of effective diagnostic methods for fetuses with chromosomal abnormalities. First, we used the BoBs™ assay to verify the overall condition of the chromosomes using a quick detection approach. Then, karyotype analysis was used to check the overall condition of chromosomes using a low-depth approach. The improved NOR-banding analysis can be utilized to detect minor changes in chromosomes, such as the short arm of chromosome 21 in case 1 of this study. Second, we used the Affymetrix CytoScan 750K Array analysis to check the deletions and shift of small segments in the chromosomes of the fetuses according to a high-depth approach. The Affymetrix CytoScan 750K Array chip contains 200,000 SNP tags and 550,000 copy number variant (CNV) tags, which are distributed throughout the whole human genome with an average density of about 1 marker/4 kb (not covering all the loci of the whole genome). It is used to detect abnormal CNVs and loss of heterozygosity (LOH), such as chromosome deletions/duplications and chromosome subtropic deletions syndrome, which are clinically significant in the whole genome. Lastly, FISH analysis is helpful for determining whether the aforementioned deletions are inherited or if new abnormalities are present.

**Figure 4:**
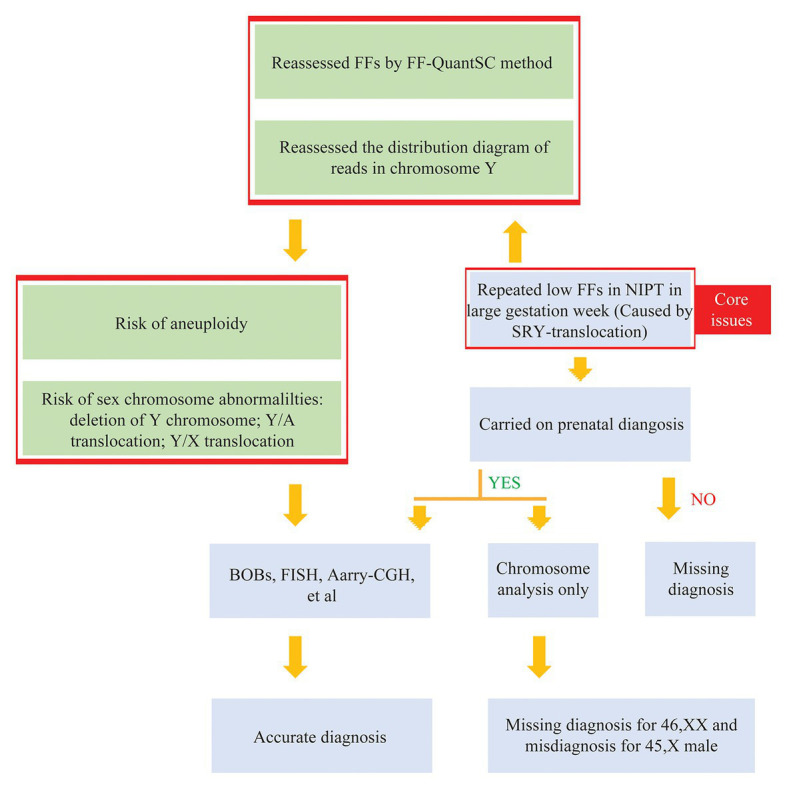
The process of disease diagnosis.

To repeat the analyses of low FF samples detected by the Y chromosome-based NIPT method for a long gestational week, we suggested a recalculation of FFs using the FF-QuantSC method and observation of the distribution diagram of reads for chromosome Y, which would aid the detection of sex chromosome abnormalities (Figure 4). In addition to the SRY translocation mentioned in this study, other chromosomal abnormalities could also be detected using FF-QuantSC, which were undetectable because of the low FF value obtained from the Y chromosome-based NIPT method. Therefore, this could prevent misdiagnosis of chromosomal abnormalities (Harris and Chan, 2019). In summary, we established a closed-loop medical system for screening and diagnosis of birth defects, which could thereby facilitate their prevention and treatment.

FF-QuantSC requires no additional sequencing data or experimental steps to estimate FF in male and female fetuses. As a result, it can be easily integrated into the routine NIPT analysis workflow without increasing its cost. Thus, FF-QuantSC is a valuable tool for FF estimation during NIPT screening, hence enhancing efficiency and reducing false-negative and false-positive rates.

## Conclusion

In this study, we focused on two fetuses with a derivative chromosome containing partial Y chromosome through various genetic diagnostic techniques, including the BoBs™ assay, karyotype analysis, improved NOR-banding analysis, Affymetrix CytoScan 750K Array, and FISH analysis. We corrected the FF by 4.1–6.0-fold using FF-QuantSC. We believe that this method is a valuable tool for the accurate estimation of FF in NIPT, especially for fetuses with a derivative chromosome containing a partial Y chromosome. For repeat low-FF NIPT cases, we highly recommend using FF-QuantSC to re-estimate FF and observing the distribution diagram of reads for chromosome Y to prevent inaccurate diagnosis.

## Data Availability Statement

The raw data supporting the conclusions of this article will be made available by the authors, without undue reservation.

## Ethics Statement

The studies involving human participants were reviewed and approved by the Hospital Ethics Committee of Shaoxing Maternity and Child Health Care Hospital and the Ethics Committee of Clinical laboratory of BGI Health. The patients/participants provided their written informed consent to participate in this study. Written informed consent was obtained from the individual(s) for the publication of any potentially identifiable images or data included in this article.

## Author Contributions

All authors listed have made a substantial, direct, and intellectual contribution to the work and approved it for publication.

### Conflict of Interest

The authors declare that the research was conducted in the absence of any commercial or financial relationships that could be construed as a potential conflict of interest.

## Supplementary Material

Supplementary Figure 1 The abnormal chromosome 21 of the fetus was not inherited from the mother in case 1.
