# Supplementary figures and images for: Application of FF-QuantSC for the Precise Estimation of Fetal Fraction in Non-invasive Prenatal Testing in Two SRY-Translocation Cases
Source: Front Genet. 2020 Oct 14;11:570333. doi: 10.3389/fgene.2020.570333 (PMC7592396; doi:10.3389/fgene.2020.570333)

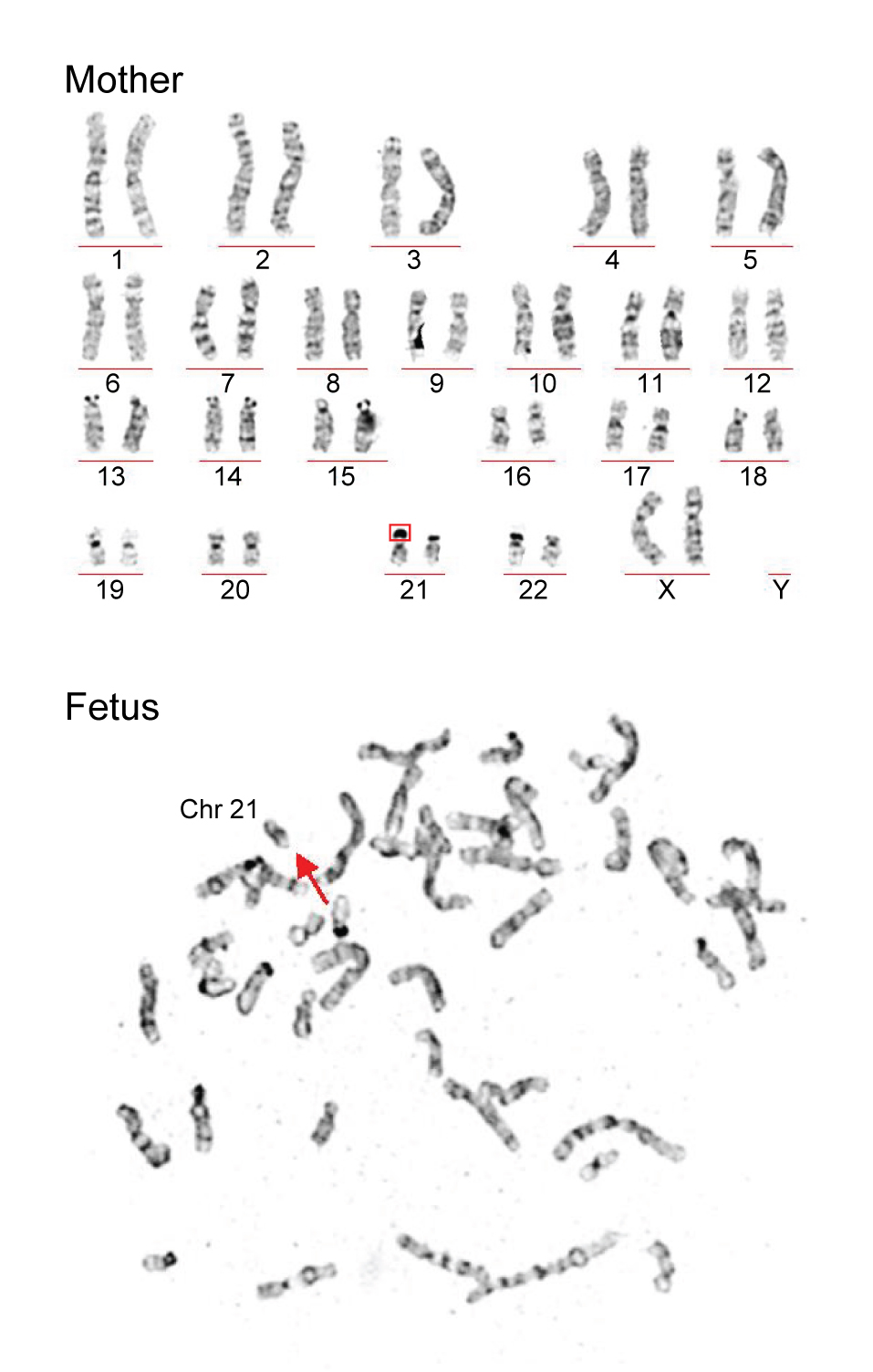

Supplement: Supplementary Figure 1 — The abnormal chromosome 21 of the fetus was not inherited from the mother in case 1. [file Image_1.jpeg]
